# Development of the Mental Synthesis Evaluation Checklist (MSEC): A Parent-Report Tool for Mental Synthesis Ability Assessment in Children with Language Delay

**DOI:** 10.3390/children5050062

**Published:** 2018-05-20

**Authors:** Julia Braverman, Rita Dunn, Andrey Vyshedskiy

**Affiliations:** 1 ImagiRation LLC, Boston, MA 02215, USA; braverju@gmail.com (J.B.); rita@engagingmath.com (R.D.); 2 Boston University, Boston, MA 02215, USA

**Keywords:** autism, ASD, psychological evaluations, ATEC, Autism Treatment Evaluation Checklist, MSEC, language delay, developmental disorder, language therapy

## Abstract

Mental synthesis is the conscious purposeful process of synthesizing novel mental images from objects stored in memory. Mental synthesis ability is essential for understanding complex syntax, spatial prepositions, and verb tenses. In typical children, the timeline of mental synthesis acquisition is highly correlated with an increasing vocabulary. Children with Autism Spectrum Disorder (ASD), on the other hand, may learn hundreds of words but never acquire mental synthesis. In these individuals, tests assessing vocabulary comprehension may fail to demonstrate the profound deficit in mental synthesis. We developed a parent-reported Mental Synthesis Evaluation Checklist (MSEC) designed to assess mental synthesis acquisition in ASD children. The psychometric quality of MSEC was tested with 3715 parents of ASD children. Internal reliability of the 20-item MSEC was good (Cronbach’s alpha >0.9). MSEC exhibited adequate test–retest reliability; good construct validity, supported by a positive correlation with the Autism Treatment Evaluation Checklist (ATEC) Communication subscale; and good known group validity reflected by the difference in MSEC scores for children of different ASD severity levels. The MSEC questionnaire is copyright-free and can be used by researchers as a complimentary subscale for the ATEC evaluation. We hope that the addition of MSEC will make the combined assessment more sensitive to small steps in a child’s development. As MSEC does not rely on productive language, it may be an especially useful tool for assessing the development of nonverbal and minimally verbal children.

## 1. Introduction

Matching words to mental images is primarily the function of Wernicke’s area, while combining images according to imposed rules is the function of the lateral prefrontal cortex (LPFC) [1,2]. The latter function of synthesizing a novel mental image from objects stored in memory is called *mental synthesis* [3]. Mental synthesis is neurologically different from other key components of imagery, such as simple memory recall and dreaming. Unlike dreaming, which is spontaneous and not controlled by the LPFC [4,5], mental synthesis is controlled by and completely dependent on an intact LPFC [6,7,8,9,10,11]. Unlike simple memory recall, which involves the recall of a single object encoded at some point in the past, mental synthesis involves the combination of two or more objects stored in memory [2,3].

Mental synthesis is defined narrowly in order to separate it from other components of executive function, such as attention, impulse control, and working memory. Mental synthesis is not a synonym of problem-solving, as complex problems can often be solved via amodal completion [12,13], spontaneous insight [14], integration of modifiers [2], and other mechanisms, that either do not require the LPFC or do not involve the combination of objects stored in memory.

Mental synthesis is highly developed in neurotypical individuals well before the age of six [15], but it is known to be a common challenge for children with Autism Spectrum Disorder (ASD). As a consequence, ASD symptoms often include a phenomenon called *stimulus overselectivity*, whereby an individual cannot mentally combine disparate objects from memory into a novel image [16,17,18]. For example, s/he will have difficulty accomplishing a seemingly trivial task, such as an instruction to “pick up a red crayon that is under the table”, which requires the individual to combine three different features, i.e., the object itself (*crayon*), its color (*red*), and its location (*under the table*). The LPFC must then mentally integrate all of these into a new mental image, *a red crayon under the table*, in order to take the correct action. When asked to *pick up a red crayon under the table*, a child with ASD who is unable to mentally synthesize the crayon with its color and location may attend to the word “crayon” and ignore both its location and the fact that it should also be red, therefore picking up any available crayon. The impaired mental synthesis affects virtually every area of an individual’s verbal, cognitive, and social functioning, including lack of comprehension of flexible syntax and spatial prepositions [19].

Furthermore, unlike vocabulary acquisition, which can be spread throughout one’s lifetime, there is only a short critical period for the development of mental synthesis capacity, since acquisition of neural networks essential for the LPFC ability to combine new images diminishes greatly after early childhood [1]. As a result, thirty to forty percent of individuals diagnosed with ASD experience lifelong impairment in the ability to understand flexible syntax and spatial prepositions [20]. These individuals, commonly referred to as having low-functioning ASD, typically exhibit full-scale IQ (intelligence quotient) below 70 [21,22] and usually perform below the score of 85 in non-verbal IQ tests (see Boucher et al. [22]; in fact, mental synthesis ability and the associated understanding of flexible syntax and spatial prepositions may be the most salient differentiator between high-functioning and low-functioning ASD).

The ASD community is very aware of this early critical period and there is wide consensus that intense early intervention should be administered to children as soon as they are diagnosed with ASD [23]. The goals of speech language pathologists (SLPs) and Applied Behavior Analysis (ABA) therapists happen to be built around the construct of mental synthesis, and therefore it happens to be highly targeted in these treatments. SLPs commonly refer to mental synthesis developing techniques as “combining adjectives, location/orientation, color, and size with nouns”, “following directions with increasing complexity”, and “building the multiple features/clauses in the sentence” [24]. In ABA jargon, these techniques are known as “visual-visual and auditory-visual conditional discrimination” [25,26,27,28], “development of multi-cue responsivity” [17], and “reduction of stimulus overselectivity” [18]. Despite the widespread recognition of the importance of early development of mental synthesis abilities, there is a lack of awareness of mental synthesis definition, its underlying neurology, and of psychometric tests that could measure a child’s progress in acquiring mental synthesis. Most language assessment tests rely heavily on a child’s vocabulary and are therefore an inadequate gauge of mental synthesis acquisition.

In this article, we present the first study of a caregiver-completed evaluation tool designed specifically to measure the acquisition of mental synthesis. This new instrument, called the Mental Synthesis Evaluation Checklist (MSEC), has been designed to complement the Autism Treatment Evaluation Checklist (ATEC) [29]. We hope that the combined ATEC/MSEC score will provide a better measure of a child’s general improvement and that the MSEC will be able to assess the function of the developing LPFC (i.e., both mental synthesis and syntactic language comprehension).

The MSEC was developed in two broad phases: (1) instrument development and (2) psychometric evaluation. The methods and results of each of these phases are described separately below.

## 2. Phase I Instrument Development

### 2.1. Methods

The purpose of the first phase, instrument development, was to create items that comprehensively capture the different facets of mental synthesis. To achieve this goal, instrument development involved three primary steps: (1) literature review; (2) concept framing; and (3) concept elicitation with patients.

#### 2.1.1. Literature Review

A literature review of publications from 1970 until 2014 was conducted in PubMed using the search terms: autism, ASD, behavioral therapy, PRT, pivotal response treatment, multiple-cue responding, conditional discrimination, stimulus overselectivity, tunnel vision, mental synthesis, cognitive therapy, language therapy with subheading and keywords applied to refine results when necessary. The purpose of this literature review was to understand the major deficits in cognitive and language development for children with ASD as it is expressed and experienced by their parents.

#### 2.1.2. Interviews with Key Opinion Leaders

Members of the research team conducted 45–60 min interviews with two psychiatrists and a clinical psychologist who treat children with ASD. Specific interview content included: treatment, challenges in caring for and communicating with children diagnosed with ASD, main concerns expressed by parents, impact of the condition on patients’ and parents’ well-being and daily functioning, use and availability of diagnostic tools and parent-reported instruments, and unmet needs within the research and patient care field.

#### 2.1.3. Concept Elicitation

The information gathered through the literature review and interviews with key opinion leaders informed an interview guide for parents of children living with ASD. This interview guide was used to conduct four parent interviews. The invitation to participate in the interview was mailed to 1217 registered users of Mental Imagery Therapy for Autism (MITA), an online educational platform designed for children with ASD [3,30,31,32], who had used it for longer than two years. The ongoing MITA trial was designed to test a tablet-based therapeutic application administered by parents to young children with ASD over the course of several years. The MITA application was developed by ImagiRation (Boston, MA, USA, www.imagiration.com) from 2013 to 2016 and made available gratis at all major app stores in February of 2016. MITA includes interactive cognitive and language puzzles designed to facilitate mental synthesis acquisition. Parents of children, users of MITA, who responded to the invitation and agreed to share their parental experience for the purposes of research were interviewed by one of the authors. The interview topics covered were: the first noticed manifestations of ASD; day-to-day experience, current interventions, methods and challenges of communication; and ways to assess the progress in a child’s development.

#### 2.1.4. Cognitive Debriefing

During the cognitive debriefing phase, we used a combination of Concurrent Think Aloud (CTA) and Retrospective Probing (RP) moderating techniques to receive appropriate feedback from caregivers regarding the MSEC preliminary items. With CTA, participants are encouraged to vocalize their thoughts as they are responding, while RP involves interviewing respondents after the session. The real-time feedback was recorded and noted by the interviewer (JB).

### 2.2. Results

#### 2.2.1. Literature Review and Interviews with Key Opinion Leaders

In typically developing individuals the faculty of mental synthesis can often be judged based on the individual’s verbal responses to a set of questions. Testing mental synthesis in nonverbal and minimally verbal children is a significantly more challenging task. Results from the literature review and key opinion leader interviews highlighted the opportunity to study the acquisition of mental synthesis from four different aspects. These aspects are described below.

##### Sentence Structure: Understanding of Noun-Adjective Combinations, Spatial Prepositions, and Flexible Syntax

The domain of sentence building is rooted in a set of common language comprehension tasks whereby a subject is required to follow verbal commands of increasing difficulty [33,34]. For example, integration of modifiers in a single object requires the subject to integrate a noun and an adjective. A subject may be asked to point to a picture of a red circle placed among several decoy images including other red shapes and circles of various colors, thus forcing the subject to notice and integrate color and object. Similarly, a subject may be asked to integrate the size descriptor and object by having to find a big circle from amongst appropriately chosen distractors. Neurologically, both integration of modifiers and mental synthesis are controlled by the LPFC, however, integration of modifiers only involves modification of neurons encoding a *single* object and, consequently, is simpler than the mental synthesis of *several* independent objects [35].

Another common task is testing the understanding of spatial prepositions such as ‘in’, ‘on’, ‘under’, ‘over’, ‘beside’, ‘in front of’, and ‘behind’ requires a subject to superimpose several objects. For example, the request to “put a green box on top of the blue box” requires an initial mental simulation of the scene, only after which is it possible to correctly arrange the physical objects. An inability to produce a novel mental image of the green box on top of the blue box would lead to the use of trial-and-error, which is likely to result in an incorrect arrangement. This instruction requires the mental combination of two objects and therefore uses the process of mental synthesis.

Syntax is a set of rules that governs the structure of sentences in a given language, particularly the word order [36,37]. A change in the word order often completely changes the meaning of a sentence (e.g., “a cat ate a mouse” vs. “a mouse ate a cat”). Understanding the precise meaning of each of these two sentences requires mental synthesis of the two objects, cat and mouse, in the proper configuration. Previous studies have demonstrated a substantial deficit in syntax comprehension among children with ASD [38].

##### Narrative Comprehension: Understanding of Stories and Explanations

The domain of narrative comprehension is rooted in a common parental activity: reading aloud to one’s child. Most parents attempt to read books to their children, however, a child who cannot imagine novel objects is unlikely to understand and follow the storyline and is therefore likely to become inattentive, a behavior which could easily be observed by a parent.

Books differ is in their complexity. Simple toddler board books filled with pictures and little text (“Goodnight Moon”, “Dear Zoo”, etc.) do not usually describe novel object combinations. Consequently, these books do not rely on mental synthesis and are generally easier to understand. More elaborate fairy tales usually contain a multitude of imaginary and hybrid objects (such as dragons and unicorns) in fantastical scenes which, since they have never been seen by the child, require mental synthesis to envision and fully comprehend the story.

##### Creative Manifestations: Drawing and Make-Believe Activities

Another window into the LPFC development of a nonverbal child is provided by the child’s creative abilities: representational drawing and make-believe activities. To generate a representational drawing, the brain must segment the object in memory into drawable fragments and then re-synthesize the fragments on paper, which is the function of the LPFC [39]. Some simpler drawings may rely on muscle memory (such as a quick doodle) and do not require mental synthesis. However, a child’s ability to draw a *novel* image following someone’s description, for example, a request to draw a three-headed dog, reveals a fully developed ability of the LPFC to mentally synthesize multiple disparate objects.

Further insights into the developing LPFC are provided by make-believe activities. Lev Vygotsky, a pioneering psychologist of the early 20th century, famously described a child who wanted to ride a horse, but since he could not, he simply picked up and straddled a stick, thus pretending that he was riding atop a horse. According to Vygotsky, a child’s relationship with the world changes around the age of three: “Henceforth play is such that the explanation for it must always be that it is the imaginary, illusory realization of unrealizable desires. Imagination is a new formation that is not present in the consciousness of the very young child, is totally absent in animals, and represents a specifically human form of conscious activity” [40]. In the child’s mind, the stick is no longer a stick; it has become a horse. The child uses mental synthesis to generate a novel experience in his/her mind: they perceive themselves riding a horse [39].

There are many other popular scenarios of make-believe activities among children, many of which seem to be universal across time and cultures. Girls between the ages of two and five often play house, picking out a doll and pretending to be its mother. For the girl, the doll becomes a live child with her own wishes and needs. To address those needs, the girl feeds the doll with imaginary food, swaddles it before putting it to sleep, dresses it in carefully selected clothes just right for the occasion, scrubs it with a sponge when it’s taking a bath, etc. The line between imaginary objects and real objects becomes quite fuzzy: dirt and grass can function as baby food; a rock can be used as a pillow [41].

Boys commonly play with trucks, tanks, and soldiers. They may pretend that the truck is a tow truck and load several smaller vehicles in the trunk. They may imagine a collision and make the cars go airborne. When playing with tanks and soldiers, the opposing armies are placed in fighting order, guns load and reload, explosions kill soldiers and make tanks explode. Most of the action, of course, is occurring solely in the mind of the child as a result of the synthesis of new, never-before-seen scenes.

##### Simple Arithmetic: Number Manipulations with Increasing Complexity

Arithmetical abilities provide an additional window into the LPFC development of a nonverbal child. The ability to understand a number as a modifier is usually acquired first. This function of the LPFC likely depends on the innate understanding of numbers by all primates [42,43] and does not rely on the mental synthesis of *multiple* objects [2]. Conversely, mental calculations requiring addition, subtraction, and multiplication are likely to rely on the mental synthesis of independent objects, especially during the initial learning phase until most single digit calculations are memorized [2].

#### 2.2.2. Existing Assessment Instruments

The assessment of the LPFC function is commonly performed with nonverbal IQ tests, such as the Test of Nonverbal Intelligence (TONI-4) [44,45], Standard Raven’s Progressive Matrices [46,47], and Wechsler Intelligence Scale for Children (WISC-V) [48]. These tests use items of progressively increasing difficulty to assess the ability of the LPFC to organize wide neuronal networks in the posterior cortex [2]. Approximately half of all questions at the beginning of each test are limited to mental computations involving only a *single* object [2]. More difficult questions located towards the end of each test rely on the mental synthesis of *several* objects [2]. Moreover, the number of objects involved in mental synthesis gradually increases with question difficulty and the LPFC is called on to organize a more widespread network of the posterior cortex [49,50,51].

Nonverbal IQ tests, however, are not available to most caregivers. Furthermore, the limited number of questions in all existing IQ tests may not be appropriate for regular testing of a child over the span of months, as the child may simply memorize the answers. Finally, nonverbal IQ tests do not measure a child’s ability to generalize mental synthesis into real-life situations. Accordingly, key opinion leaders indicated that the appropriate parent-administered tool to identify mental synthesis acquisition was absent from existing Patient Reported Outcome measures and may be important to identify specific aspects of the development deficit of children with ASD.

#### 2.2.3. Conceptual Framing

Results from the literature review and key opinion leader interviews highlighted the importance of evaluating four different facets of mental synthesis, such as (1) Sentence structure: understanding noun-adjective combinations, spatial prepositions, and flexible syntax; (2) Narrative comprehension: understanding of stories and explanations; (3) Creative manifestations: drawing and make-believe activities; and (4) Simple arithmetic: number manipulations with increasing complexity.

**Figure 1:**
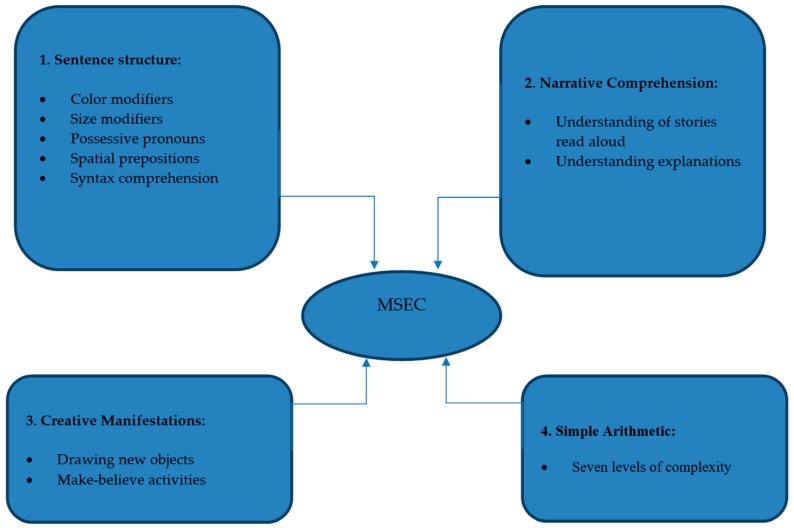
MSEC (Mental Synthesis Evaluation Checklist) preliminary conceptual map.

Figure 1 depicts the preliminary conceptual map constructed based on the results of the literature review and qualitative analysis of the interviews with key opinion leaders.

#### 2.2.4. Concept Elicitation

Seventy-two parents responded to the MITA usability survey. Thirty-seven of those parents (51%) agreed to be contacted for a follow-up interview and to share their experience as a parent of a child living with ASD. Ten parents (randomly selected from thirty-seven responders) were interviewed for the concept elicitation stage. Parents’ feedback provided additional support for the preliminary conceptual frame that was built based on the literature review and the key opinion leaders interviews. Specifically, parents indicated difficulties in ascertaining the exact level of their child’s comprehension. The thematic content analysis identified specific everyday parent-child activities that potentially involve LPFC in each of the domains described above. These activities were used to generate 20 items used in the preliminary version of the MSEC.

#### 2.2.5. Item Generation

Item generation was informed by the results from all previous stages of data collection, i.e., literature review and interviews with key opinion leaders and caregivers. The goal was to develop a comprehensive evaluation to ensure construct validity of the measured concept: mental synthesis acquisition. For each domain, we initially aimed to assess the simpler function of the LPFC related to the modification of a *single* object and then to proceed to the assessment of mental synthesis of *multiple* objects—the most complex function of the LPFC [35]. For example, the first three items in the Sentence structure domain assess the simpler function of the LPFC, such as integration of a color and size modifier, and the last four items assess mental synthesis of multiple objects (see Table 1). Similarly, the first item in each of the other three domains assesses the simpler function of the LPFC related to the modification of a *single* object while the following items assess the mental synthesis of *multiple* objects.

We considered the caregiver response burden, aiming to shorten the time needed to complete the assessment using the tool described above. The wording of items was informed specifically by the language used by caregivers during the interviews and was driven conceptually by children’s and caregivers’ everyday experience. Finally, we structured the items to be applicable to nonverbal children. Table 1 shows the preliminary list of items with the corresponding domains. For each item, a caregiver was asked how much each of the following was true regarding his/her child on a scale: not true, somewhat true, and very true.

#### 2.2.6. Cognitive Debriefing

Three caregivers (two males) were interviewed during the cognitive debriefing phase. Their children were 4–5 years old, diagnosed with moderate to severe ASD. The mean time required to complete the MSEC was less than 5 min, indicating a low response burden. The general impression of the MSEC was positive. All participants indicated that the arithmetic questions (except ‘understanding of numbers’) were not yet relevant to their children (they selected the response option “not true” for all of them) but may be relevant for older children. Two participants noticed minor difficulties in interpreting item 1 (“understanding simple modifiers”), indicating that their child understands “some modifiers, but not others”. One participant noted that the meaning of “elaborate story” (item 9) is “subject to interpretation”. One participant had difficulty understanding item 11. Based on this qualitative analysis of caregiver’s feedback, a number of items were revised to improve clarity. Table 1 presents the modified 20–item MSEC that was administered to a new sample of caregivers for psychometric evaluation.

## 3. Phase II. Psychometric Validation

### 3.1. Methods

#### 3.1.1. Data Collection

Twenty items of the preliminary MSEC were administered online to parents of children diagnosed with ASD, who were also participants in the Mental Imagery Therapy for Autism (MITA) observational trial [30,31,32,52]. Approximately one month after the first use of MITA and no sooner than 100 puzzles had been solved, parents were required to complete the informed consent, the 20-question MSEC, and the Autism Treatment Evaluation Checklist (ATEC) [29]. Subsequently, parents were asked to complete MSEC and ATEC at three-month intervals in order to continue their use of MITA. Both evaluations were completed inside the MITA application on the same device that was used to administer MITA.

#### 3.1.2. Participants

From the pool of 42,145 registered MITA users, we selected 3724 participants who had completed at least two evaluations. All but 63 participants completed the evaluations on several different dates. Of the 63 participants who completed more than one evaluation on the same day, nine were removed from the analysis for obtaining highly discrepant scores on two or more same-day evaluations (i.e., Total ATEC score SD ≥ 25.3 or MSEC SD ≥ 8.7). The scores of the remaining 54 participants who completed two or more evaluations on the same day, and received similar scores, were averaged. The final sample contained 3715 participants. The self-reported median age of participants was 4 (range from 1 to 12 years old, 71% males). The majority of subjects (63%) resided in the USA.

#### 3.1.3. Measurements

The ATEC is a 77-item measurement tool, it is designed to be completed by parents, teachers, or caretakers, and has been validated in a number of studies [53,54,55,56,57]. It consists of four subscales: I. Speech/Language/Communication (14 items); II. Sociability (20 items); III. Sensory/Cognitive Awareness (18 items); and IV. Health/Physical/Behavior (25 items). The ATEC was designed for use by caretakers and educators to monitor how well a child is doing over time [29]. In addition, researchers have used the ATEC to document improvements following an intervention by comparing the baseline ATEC scores with the post-treatment ATEC scores [58].

The preliminary 20-item version of the MSEC required parents to rate their child’s habits and abilities using a 0 (not true) to 2 (very true) Likert-type rating scale.

As a part of the current study, demographic information about sex, age, diagnosis, and the first diagnosis date of participants’ children was collected from their registration data. 

### 3.2. Results

The factor analysis revealed that one eigenvalue of the polychoric correlation is substantially higher than the rest (the maximum eigenvalue of 11.84 compared to the next highest eigenvalue of 2.87). This confirms the reasonable unidimensionality of the scale. A confirmatory factor analysis revealed a strong correlation (>0.7) of all items with the single factor. All the seven items in the Simple arithmetic domain showed a substantial floor effect (i.e., >75% of “not true” responses), which may be due to relatively severe ASD level or young age of participants in our sample. Despite the strong floor effect for items in the Simple arithmetic domain, all items’ actual response ranges matched with their theoretical ranges.

#### 3.2.1. Reliability

Internal consistency was excellent (Cronbach’s alpha equals 0.93), suggesting high reliability. All items demonstrated high (>0.4) item-total correlations. The MSEC test–retest reliability was evaluated by calculating a Pearson Correlation between the first administration of the MSEC and the re-administration of the MSEC to the same participants approximately 3 months (83–96 days) and 9 months (260–288 days) later. Only participants who reported a stable ATEC score (i.e., stable ASD condition) were included in the analysis. The ATEC score was considered stable if the discrepancy between two evaluations was no more than 10 points. Two hundred and forty-one (241) participants had stable ATEC scores for evaluations completed 3 months apart and 46 participants had stable ATEC scores for evaluations completed 9 months apart. The 3-month test–retest correlation coefficient for MSEC was *r* = 0.66 (*p* < 0.001), and the 9 months test–retest correlation coefficient of MSEC had *r* = 0.6 (*p* < 0.001), revealing moderate MSEC long-term stability.

#### 3.2.2. Validity

To evaluate convergent and discriminant validity, a correlation matrix of the MSEC and all ATEC subscales was constructed. The MSEC was positively correlated with Communication subscale of ATEC (*r* = 0.6, *p* < 0.01), which testified to its convergent validity. The correlation with other subscales and the ATEC total score was low (r ranged from 0 to 0.43, with *r* = 0.28 for the ATEC total score), suggesting the uniqueness of the concept measured by MSEC compared to the ATEC total score and its subscales other than the Communication subscale.

To evaluate known-groups validity, we calculated average MSEC scores for four groups determined by the level of ASD severity based on the ATEC total score. Table 2 shows MSEC scores for Mild (ATEC total score ≤ 49), Moderate (49 > ATEC total score ≤ 80), and Severe (ATEC total score ≥ 80) groups. The ANOVA with contrasts demonstrated significant difference between all groups: F (2, 3712) = 142.23, *p* < 0.001. All differences were in the expected direction (i.e., lower MSEC scores were reported for milder ASD groups).

## 4. Discussion

In typical children, Wernicke’s area develops concurrently with the lateral prefrontal cortex (LPFC), but in children with ASD, development of one cortical area can significantly outpace the other. Commonly, the development of Wernicke’s area is significantly faster than the development of the LPFC and, as a result, understanding of words significantly outpaces acquisition of mental synthesis and its dependent functions such as understanding of flexible syntax and spatial prepositions [19,22,59,60]. In these children, whose Wernicke’s area and LPFC are developing asynchronously, we ought to measure their functions separately. It is not uncommon to observe the following developmental steps in children who acquire language with a significant delay: they start to understand some individual words and phrases (Wernicke’s area), then develop understanding of more complex syntactic language (LPFC), and only after that they begin to verbally express themselves, first with individual words and then with complete sentences (Broca’s area). The existing tests and evaluations adequately assess the former (receptive vocabulary acquisition) as well as the latter (expressive language development), but, critically, miss assessing the middle step, which heralds the LPFC function of mental synthesis and the corresponding understanding of syntactic language. Therefore, there is a substantial gap in the ability of these tests to faithfully measure a child’s developmental progress. To fill this gap, the Mental Synthesis Evaluation Checklist (MSEC) was developed. Its design closely adhered to the recommendations of the US Food and Drug Administration patient-reported-outcomes development guide [61]. MSEC consists of 20 items that capture four aspects of mental synthesis acquisition from the perspective of caregivers of children with ASD: (1) Sentence structure: understanding noun-adjective combinations, spatial prepositions, and flexible syntax; (2) Narrative comprehension: understanding of stories and explanations; (3) Creative manifestations: drawing and make-believe activities; and (4) Simple arithmetic: number manipulations with increasing complexity.

Information collected from the literature, key opinion leaders, and caregivers helped to inform a scientifically grounded and relevant measure of mental synthesis in children with ASD. Although a rigorous qualitative approach was given to elicit and to collect caregivers’ input throughout the initial steps of instrument development, some limitations in sample selection should be noted. We only interviewed a sample size of caregivers who volunteered to share their experience. This may have caused some selection bias and affected generalizability of the new instrument. Future studies should further validate its use with parents of various subcategories of ASD children. Despite this limitation, the new instrument demonstrated good psychometric qualities. The factor analysis confirmed unidimensionality of the test items, implying that all the items measure a single concept. The MSEC test exhibited good internal consistency and adequate test–retest reliability for patients with stable ASD level after follow-up periods of 3 and 9 months. The construct validity of the MSEC was confirmed by its positive correlation with the ATEC Communication subscale. The MSEC scores were significantly different for children with different ASD severity levels, confirming the construct validity of the new instrument. The MSEC may be conveniently used as a compliment to existing measures of ASD (e.g., ATEC).

Although the current empirical evaluation demonstrated a strong evidence of good psychometric properties, such as validity and internal consistency, there are some noteworthy limitations. The study population has been selected from registered users of MITA, which may have introduced some bias toward technologically advanced caregivers. Test–retest reliability was good although not excellent. This is likely because the time gap between test administrations was too long, as evaluations were administered with a periodicity of three months. Future studies should be conducted to recheck and improve the test–retest reliability of MSEC.

The MSEC questionnaire described in this manuscript is copyright-free and can be used by researchers as a complimentary subscale for the Autism Treatment Evaluation Checklist. We hope that the addition of MSEC to existing evaluations will make the combined assessment less punctured and more sensitive to small steps in a child’s development. As MSEC does not rely on productive language, it may be an especially useful tool for assessing the development of nonverbal and minimally verbal children.

## 5. Compliance with Ethical Standards

All procedures performed in studies involving human participants were in accordance with the ethical standards of the institutional and/or national research committee and with the 1964 Helsinki declaration and its later amendments or comparable ethical standards. This observational study is exempted from institutional review board and informed consent according to Code of Federal Regulations, TITLE 45, PUBLIC WELFARE, DEPARTMENT OF HEALTH AND HUMAN SERVICES, PART 46, PROTECTION OF HUMAN SUBJECTS, Subpart A, Basic HHS Policy for Protection of Human Research Subjects, §46.101 (b) (2).

## Figures and Tables

**Table 1:** Preliminary list of MSEC items. For each item, a caregiver is asked how much each of the following is true regarding his/her child on a scale: not true, somewhat true, and very true.

	My Child	Domain of Mental Synthesis
1	Understands some simple modifiers (i.e., green apple vs. red apple or big apple vs. small apple)	Sentence structure: understanding of noun-adjective combinations, spatial prepositions, and flexible syntax
2	Understands several modifiers in a sentence (i.e., small green apple)	
3	Understands size (can select the largest/smallest object out of a collection of objects)	
4	Understands possessive pronouns (i.e., your apple vs. her apple)	
5	Understands spatial prepositions (i.e., put the apple ON TOP of the box vs. INSIDE the box vs. BEHIND the box)	
6	Understands verb tenses (i.e., I will eat an apple vs. I ate an apple)	
7	Understands the change in meaning when the order of words is changed (i.e., understands the difference between “a cat ate a mouse" vs. “a mouse ate a cat”)	
8	Understands simple stories that are read aloud	Narrative comprehension: understanding of stories and explanations
9	Understands elaborate fairy tales that are read aloud (i.e., stories describing FANTASY creatures)	
10	Understands explanations about people, objects, or situations beyond the immediate surroundings (e.g., “Mom is walking the dog,” “The snow has turned to water”)	
11	Draws a VARIETY of RECOGNIZABLE images (objects, people, animals, etc.)	Creative manifestations: drawing and make-believe activities
12	Can draw a NOVEL image following YOUR description (e.g., a three-headed animal)	
13	Engages in a VARIETY of make-believe activities (such as: playing house, playing with toy soldiers, building forts, and castles, etc.)	
14	Understands NUMBERS (i.e., two apples vs. three apples)	Simple arithmetic: number manipulations with increasing complexity
15	Can perform simple arithmetic: 2 + 3 = ?	
16	Can add larger numbers: 7 + 6 = ?	
17	Can perform simple subtraction: 3 − 2 = ?	
18	Can subtract larger numbers: 15 − 7 = ?	
19	Can perform simple multiplication: 2 × 2 = ?	
20	Can multiply larger numbers: 6 × 7 = ?	

MSEC: Mental Synthesis Evaluation Checklist.

**Table 2:** MSEC scores as a function of ASD severity.

ASD Severity	*n* (%)	MSEC	
		Mean (SD)	Contrast F for Adjacent Groups
Mild	636 (17%)	24.89 (7.96)	194.53 (Mild vs. Moderate; *p* < 0.001)
Moderate	1586 (43%)	30.23 (7.23)	13.00 (Moderate vs. Severe; *p* < 0.001)
Severe	1493 (40%)	31.29 (9.10)	

ASD: Autism Spectrum Disorder.
